# Androgen Deprivation Freezes Hormone-Sensitive Prostate Cancer Cells in a Reversible, Genetically Unstable Quasi-Apoptotic State, Bursting into Full Apoptosis upon Poly(ADP-ribose) Polymerase Inhibition

**DOI:** 10.3390/ijms24032040

**Published:** 2023-01-20

**Authors:** Andrea Pelliccia, Francesco Capradossi, Francesca Corsi, Greta Deidda Tarquini, Emanuele Bruni, Albrecht Reichle, Francesco Torino, Lina Ghibelli

**Affiliations:** 1 Department of Biology, University of Rome “Tor Vergata”, 00133 Rome, Italy; 2 Department of Chemical Science and Technologies, University of Rome “Tor Vergata”, 00133 Rome, Italy; 3 Department of Internal Medicine III, Hematology and Oncology, University Hospital of Regensburg, 93053 Regensburg, Germany; 4 Department of Systems Medicine, Medical Oncology, University of Rome “Tor Vergata”, 00133 Rome, Italy

**Keywords:** hormone-sensitive prostate cancer, androgen deprivation therapy, apoptosis, PARP, quasi-apoptotic state

## Abstract

Androgen deprivation therapy (ADT) is a powerful treatment for metastatic hormone-sensitive prostate cancer (mHSPC) patients, but eventually and inevitably, cancer relapses, progressing to the fatal castration-resistant (CR)PC stage. Progression implies the emergence of cells proliferating in the absence of androgen through still elusive mechanisms. We show here for the first time that ADT induces LNCaP mHSPC cells to collectively enter a metastable quasi-apoptotic state (QUAPS) consisting of partial mitochondrial permeabilization, limited BAX and caspase activation, and moderate induction of caspase-dependent dsDNA breaks; despite this, cells maintain full viability. QUAPS is destabilized by poly(ADP)-polymerase inhibition (PARPi), breaking off toward overt intrinsic apoptosis and culture extinction. Instead, QUAPS is rapidly and efficiently reverted upon androgen restoration, with mitochondria rapidly recovering integrity and cells collectively resuming normal proliferation. Notably, replication restarts before DNA repair is completed, and implies an increased micronuclei frequency, indicating that ADT promotes genetic instability. The recovered cells re-acquire insensitivity to PARPi (as untreated LNCaP), pointing to specific, context-dependent vulnerability of mHSPC cells to PARPi during ADT. Summarizing, QUAPS is an unstable, pro-mutagenic state developing as a pro-survival pathway stabilized by PARP, and constitutes a novel viewpoint explaining how ADT-treated mHSPC may progress to CRPC, indicating possible preventive countermeasures.

## 1. Introduction

Androgen deprivation therapy (ADT), consisting of chemical or surgical castration, which is associated with androgen receptor (AR) signaling inhibition (ARSI) and possibly chemotherapy [1], is the present main therapeutic approach for metastatic hormone-sensitive prostate cancer (mHSPC) that efficiently promotes tumor control/regression [2]. However, unfortunately the disease almost inevitably progresses/relapses due to the appearance of cells capable of replicating despite castration (castration-resistant PC, CRPC), leading to patients’ death [1].

Initial tumor response implies ADT-promoted cell death, occurring mainly due to apoptosis, as indicated in many retrospective studies on patients’ specimen [3]. However, in vitro modelling has questioned the occurrence of ADT-induced apoptosis [4]. Indeed, it has been suggested that the occurrence of apoptosis in HSPC cells under ADT may not derive from a direct effect of castration on PC cells but rather, from tissue-related mechanisms indirectly impairing PC cell survival. It has indeed been shown that cancer-associated fibroblasts present in stroma contribute to epithelial cell apoptosis via metalloproteinase activation [5]; moreover, ADT causes reduced blood flow due to the impairment of prostate vessels [6].

Multiple mechanisms of ADT resistance have been established as contributors to the progression of HSPC to CRPC. Remarkably, along with intra-tumoral and alternative androgen production, AR-dependent pathways remain key drivers in this progression, and include the amplification or gain of function mutations in AR, the development of functional splice variants, post-translational regulation and pro-oncogenic modulation in the expression of coactivators vs. corepressors of AR [7]. More recently, other pathways have been recognized as “ancillary” factors in the progression from HSPC to CRPC, such as RAS/MAP kinases, TGF-β/SMAD pathway, FGF signaling, JAK/STAT pathway, Wnt-βcatenin and hedgehog signaling, cell adhesion molecules, G-protein-coupled receptors and some miRNAs as well [8].

The in vitro/in vivo ADT models allow the observation of the very early events occurring in PC under androgen deprivation. These consist in the immediate arrests of PC cell proliferation, induction of senescence [9] and promotion of epithelial-to-mesenchymal transition (EMT), without notable cell death [10,11,12]. However, if and how such events lead, in the long run, to gene expression reprogramming and ultimately to the emergence of castration-resistance, remains unknown.

To address the still obscure mechanisms behind the escape from ADT, we performed in vitro experiments on the well-known HSPC LNCaP cell line upon androgen deprivation; we confirmed cytostasis, senescence and EMT, but we also discovered that ADT immediately and collectively freezes all cells in a metastable quasi-apoptotic state (QUAPS), where partial mitochondrial permeabilization causes limited caspase activation, leading to moderate DNA breaks. Notably, the inhibition of poly(ADP-ribose) polymerase (PARP), a therapeutic protocol often used in clinics to sensitize cancer cells to apoptosis [13], breaks-off QUAPS into overt apoptosis, extinguishing the culture. Vice versa, androgen restoration “heals” cells in QUAPS, promoting a collective, rapid return to proliferation, at the same time losing sensitivity to PARPi. Healing implies DNA repair, which however occurs after the restoration of proliferation, leading to substantial mutagenesis.

These results propose a totally new viewpoint, pointing to ADT-promoted genetic instability as a main factor of progression.

## 2. Results

### 2.1. ADT Induces Immediate Cytostasis in the Hormone-Sensitive PC Cells LNCaP

**Figure 1:**
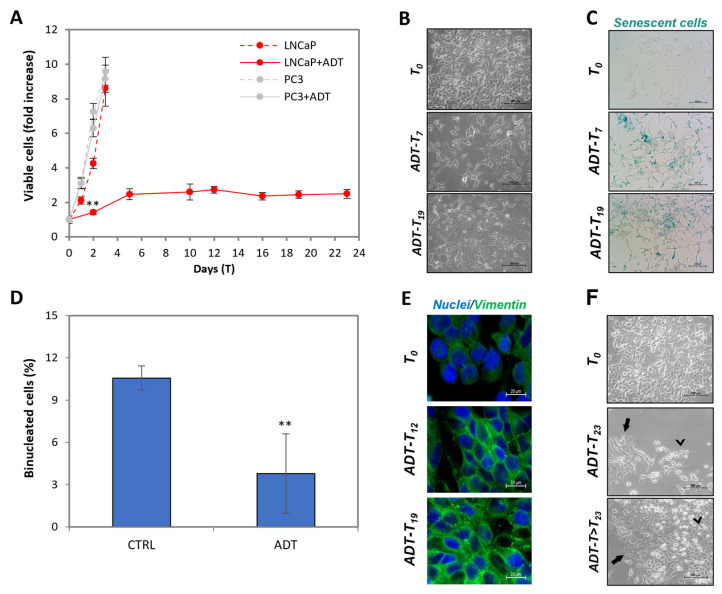
ADT induces cytostasis in the hormone-sensitive LNCaP cells. (**A**) Kinetics of viable (trypan blue-excluding) cell number expressed as fold over T0. Dashed lines indicate control cells. Statistical significance was calculated via Student’s *t*-test. ** *p* < 0.01, with respect to control cells. (**B**) Phase-contrast images of LNCaP during ADT at the indicated time points. The scale bar corresponds to 200 µm. (**C**) β-galactosidase staining at T0, T7 and T19. The scale bar corresponds to 200 µm. (**D**) Frequency of bi-nucleated cells at T5 of ADT with respect to control cells. Statistical significance was calculated via Student’s *t*-test. ** *p* < 0.01 respect to control cells. (**E**) Immunofluorescence with anti-vimentin antibody (green) and DAPI (blue) in wide-field micrography. The scale bar corresponds to 20 µm. (**F**) Phase-contrast images of LNCaP after ≥ 23 days of ADT. Black arrows indicate cells surviving ADT, arrow heads indicate ADT-resistant clones. The scale bar corresponds to 200 µm.

We employed the HSPC-derived LNCaP cell model to reproduce in vitro the events occurring upon ADT. Cells were cultured in androgen-free medium to mimic radical castration. Figure 1A shows that ADT gradually slows down LNCaP proliferation, to reach complete cytostasis at T5. To note, proliferation of CRPC PC3 cells is totally insensitive to ADT. LNCaP cytostasis is associated with very evident changes in morphology, as shown in Figure 1B: cells first acquire a vaguely star-like shape (T7), then form thread-like structures, that are very closely associated (see T19). Notably, the state of semi-confluence is only slightly affected. The proliferative quiescence is confirmed by the acquisition of senescence markers (Figure 1C) and cell cycle arrest, as shown by the low number of binucleated cells when cultured in the presence of the cytokinesis inhibitor cytochalasin B (Figure 1D). Figure 1E shows the overexpression of epithelial-to-mesenchymal transition (EMT) markers, such as vimentin. Notably, apoptosis is not an immediate result of ADT (see later), confirming general observations, especially in in vitro ADT modeling. All these features, as previously described [9,10,11,12], represent a validation of our model system.

Figure 1F shows that, on some rare occasions (2 out of 40), 23–30 days after ADT, clones of cells begin to proliferate in the absence of androgen (see arrow indicating initial clonal growth in the picture). Notably, the resulting cells, which we could stabilize and study thoroughly as a new cell line, lost most of the parental LNCaP morpho-functional features to acquire those typical of CRPC cells (work in progress). This further validates our system as a bona fide model of what occurs in HSPC patients treated with ADT and progressing to CRPC.

During prolonged (i.e., months) exposure to ADT, cells become rarer and rarer; however, this never leads to culture extinction for periods as long as 6 months, rather stabilizing at about 1/20 of the initial number. Notably, the survivors acquired a rounded appearance resembling what has been reported by others [14], suggesting the acquisition of a dormant [15] phenotype.

### 2.2. ADT Activates the Intrinsic Apoptotic Pathway without Affecting Cell Viability

Androgen deprivation may induce apoptosis on prostate cells [16]; therefore, we looked for apoptotic features in cells in ADT.

The intrinsic, stress-induced apoptotic pathway begins with the activation of BAX (exposure of the N-term detectable with the 6A7 antibody) that translocates to the mitochondria causing the dissipation of transmembrane potential (mtΔΨ) and cytochrome c release in the cytosol, where it organizes the apoptosome to activate the apical caspase-9, which in turn activates caspase-3 that finally organizes cell dismantling through its proteolytic action, acting on many direct protein targets and leading to canonical features such as nuclear vesiculation, PS exposure and internucleosomal DNA endo-digestion [17].

**Figure 2:**
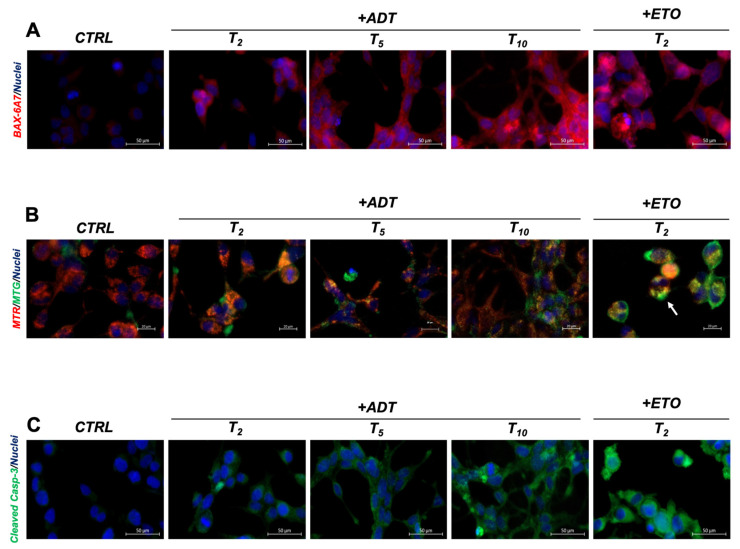
ADT activates the intrinsic apoptotic pathway without affecting cell viability. (**A**) ADT induces time-dependent sub-apoptotic activation of BAX. Immunofluorescence with anti-activated-BAX (6A7) antibody (red) and DAPI (blue) for nuclei, in wide-field micrography. The scale bar corresponds to 50 µm. (**B**) ADT alters the mitochondrial activity in the whole cell population. The prevalent red pattern of untreated cells (T0) turns into a prevalent orange/yellow pattern during ADT. Live cells staining of the mitochondrial mass (MTG, green), mitochondria with high transmembrane potential, mtΔΨ (MTR, red) and cell nuclei (Hoechst, blue) in wide-field micrography. The scale bar corresponds to 20 µm. (**C**) ADT increases caspase-3 activation in the whole cell population. Immunofluorescence with anti-cleaved caspase-3 antibody (green) and DAPI (blue) for nuclei, in wide-field micrography; the strong green signals correspond to apoptotic cells. The scale bar corresponds to 50 µm.

We investigated BAX activation, as shown in Figure 2A, where LNCaP cells stained with the 6A7 antibody are analyzed. The increased BAX staining shows that BAX is activated during ADT in a time-dependent fashion, suggesting that mitochondria are permeabilized. Notably, this occurs in all treated cells, albeit at a reduced level, with respect to what occurs in the positive controls of apoptosis, such as high doses of etoposide. In this case, apoptosis is not a collective phenomenon, but rather, a scattered event, and apoptotic cells co-exist with cells either in a viable or pre-apoptotic state.

We then analyzed the mitochondrial transmembrane potential (mtΔΨ), which drops upon BAX translocation and activation in apoptosis [18]. Figure 2B shows the fluorescence micrograph of mitochondria in un-fixed cells, co-stained with MitoTracker Red (MTR, staining only mitochondria with high mtΔΨ) and MitoTracker Green (MTG, staining the mitochondrial mass irrespective of mtΔΨ). The prevalent red pattern of untreated cells (T0) turns into a prevalent orange/yellow pattern during ADT, indicating the co-existence of charged and uncharged mitochondria. The mitochondria alterations occur in the whole cell population, i.e., they are not limited to a subset of cells, indicating a collective cell reaction synchronously occurring upon hormone deprivation. Additionally, in this case, the levels of mitochondrial alterations do not reach what is found in overt apoptosis, where the color pattern is exclusively green (only uncharged mitochondria present), as evident in the scattered apoptotic cells present in the culture of the apoptosis-positive controls (arrows).

We next analyzed caspase-3, observing that it is also activated by ADT, being detectable in situ by cleaved-caspase-3-specific antibodies, which delineate a diffused pattern that increases with the time of ADT (Figure 2C). Caspase activation encompasses all cells, and never reaches the levels found in full apoptosis, as evidenced in etoposide-treated cells.

Such ADT-induced alterations do not correspond to full apoptosis, as revealed by the maintenance of LNCaP viability (measured as fraction of trypan blue negative cells), over 95% for all the time points considered (up to T23), and by the lack of detectable apoptosis (e.g., nuclear vesiculation) at all the time points considered (see also later). Importantly, the fact that all cells share such alterations, indicate that they are responding to specific signals rather than being the result of damage.

Overall, these data show that the main actors of the intrinsic apoptotic pathway, i.e., that induced by stress, are partially activated in all cells in ADT, but nonetheless, cells maintain total viability.

### 2.3. ADT Immediately Induces Caspase-Dependent dsDNA Breaks

Since a main target of caspases is iCAD, the endogenous inhibitor of the CAD endonuclease responsible for apoptotic DNA digestion [19], we explored whether the partial apoptotic features induced by ADT would include DNA endo-digestion as a consequence of caspase activation.

**Figure 3:**
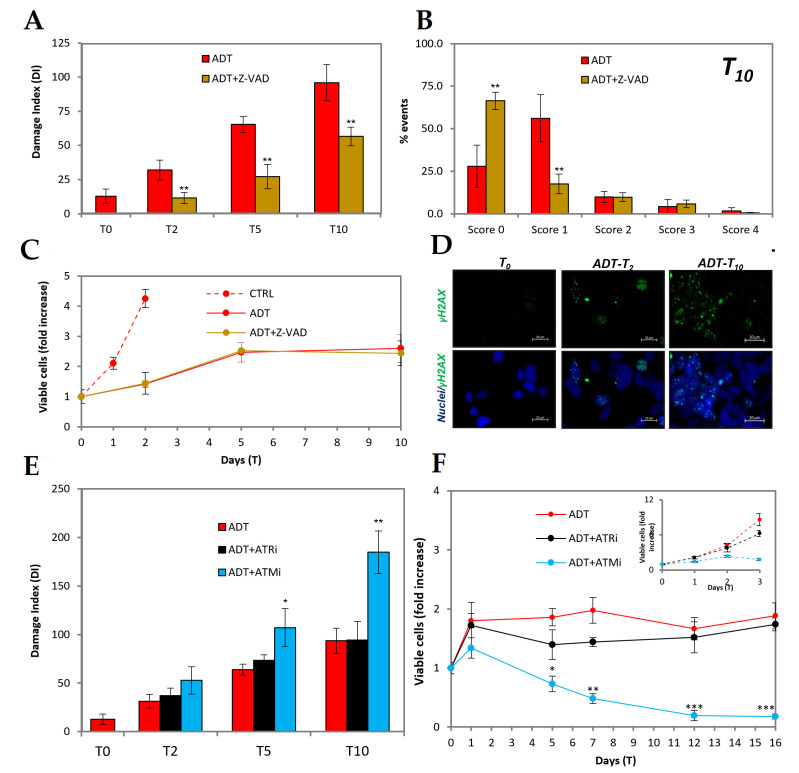
ADT induces caspase-dependent dsDNA breaks in mHSPC LNCaP cells. (**A**) Comet assay analysis of ADT-treated LNCaP ± Z-VAD: the histogram reports the Damage Index at the indicated time points. (**B**) The histogram shows the scores (from 0 to 4) of DNA damage of ADT-treated LNCaP in presence/absence of Z-VAD at the indicated time point. (**C**) Kinetics of viable (trypan blue-excluding) cell number expressed as fold over T0, showing that caspase inhibition does not affect ADT-treated LNCaP nor control LNCaP survival. (**D**) Immunofluorescence with anti-γH_2_AX antibody and DAPI for nuclei in wide-field micrography. The scale bar corresponds to 20 µm. (**E**) Comet assay of ADT-treated LNCaP in presence/absence of ATM or ATR inhibitors: the histogram reports the Damage Index at the indicated time points. (**F**) Kinetics of viable (trypan blue-excluding) in ADT ± ATM or ATR inhibitors; cell number expressed as fold over T0. The inset reports the effect of ATM/ATR inhibitors on control cells. Statistical significance was calculated via Student’s *t*-test. * *p* < 0.05, ** *p* < 0.01 and *** *p* < 0.001, with respect to ADT-treated cells.

Figure 3A shows that very early in ADT DNA breaks form, as evidenced by the comet assay signal, which is significantly higher than the control already at T2. The signal increases afterwards (red bars) to reach at T10 a level only 3-fold lower than that induced by 50uM etoposide, a topoisomerase II poison directly producing double-stranded DNA breaks. Notably, the pan-caspase inhibitor z-VAD (Figure 3A, brown bars) prevents ADT-induced DNA damage at T2, showing that it is an event self-induced due to caspase activation. At later time points, the comet signal raises in ADT-treated cells also in the presence of z-VAD, suggesting that other signals may intervene, such as, e.g., the AIF/endoG pathway, which also depends on mitochondrial permeabilization [20]. However, the DNA damage level remains significantly and strongly below the corresponding time points of ADT.

In order to discriminate whether the DNA damage score is due to few cells with high damage or many cells with low damage, we developed the analysis of the comet data in the different classes of damage at T10. Figure 3B shows that most cells belong to the non-damaged (or damaged below detection?) or mildly damage classes, whereas the instances of highly damaged cells are as low as in the controls. Therefore, as for the other moderate apoptotic features described in the previous chapter, DNA damage is shared by most cells, remaining at low level in each of them. Notably, the inhibition of caspase-mediated DNA damage does not influence the kinetics of cell number in ADT-treated cells (Figure 3C), confirming the general assumption that the growth arrest induced by hormone withdrawal is achieved independently from the DNA damage response.

The damage is perceived by cells as a double-strand DNA damage, since it is able to engage a DNA damage response as shown by the activation of the nuclear ɣH_2_AX signal (Figure 3D).

It is well-known that CAD produces double-strand (ds) DNA breaks that are essentially blunt-ended [19], and should elicit activation of the DNA repair machinery via ataxia telangiectasia mutated (ATM) kinase, which is the chief coordinator of the DNA damage response, responsible for organizing the repair of dsDNA breaks [21]. ATM inhibition therefore would enhance the extent of dsDNA breaks by impairing their repair. Indeed, ATM inhibition greatly increases (nearly 2-fold) ADT-induced DNA breaks, as shown in Figure 3E, by comet assay. On the contrary, the inhibition of ATM and RAD3-related (ATR), the kinase coordinating response and the single-strand (ss) DNA breaks repair, does not interfere with ADT-induced DNA damage.

Accordingly, ATM inhibition is accompanied by a strong cytotoxic effect resulting in drop of the number of viable cells as early as after 5 days of ADT (Figure 3F), whereas the inhibition of ATR does not substantially affect ADT-induced cytostasis (Figure 3F).

Overall, this set of data shows that ADT promotes caspase-mediated DNA damage producing double-stranded breaks, which must be at least in part repaired by the ATM-signaling machinery to maintain cell viability.

### 2.4. PARP Inhibition Promotes Full Apoptosis in ADT-Treated LNCaP

It was reported that PARPi is synthetically lethal in ADT-treated cells, but not in untreated HSPC cells [22], though the mechanism remains obscure. To explore the issue, we used two inhibitors of PARP, namely, the nicotinamide mimetic 3-aminobenzamide (3ABA), well-known in our lab [13,23,24], and olaparib (OLA), a clinically relevant new-generation-specific PARP poison [10], on LNCaP cells in ADT. 

**Figure 4:**
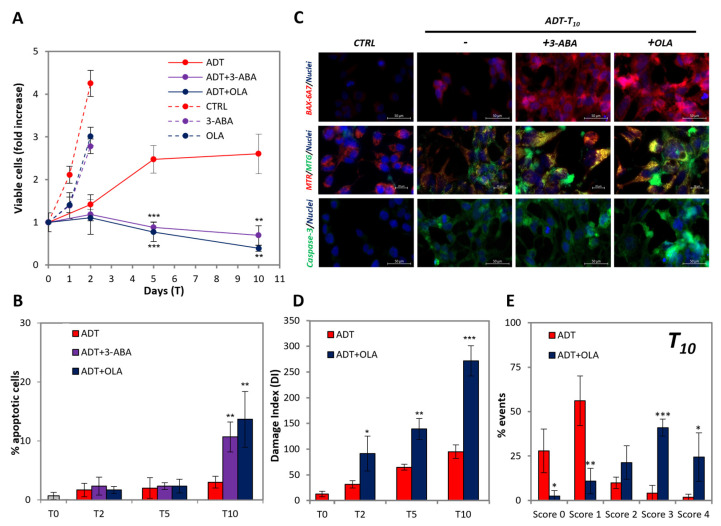
PARP inhibition promotes full apoptosis in ADT-treated LNCaP. (**A**) Kinetics of viable (trypan blue-excluding) cell number in ADT ± PARP inhibitors in LNCaP expressed as fold over T0. Dashed lines indicate control cells ± 3-ABA/OLA. (**B**) Extent of apoptosis in LNCaP exposed to ADT ± PARP inhibitors at the indicated time points. Statistical significance was calculated via Student’s *t*-test. **p < 0.01 and *** *p* < 0.001, with respect to ADT-treated cells. (**C**) ADT-promoted BAX activation (top line), mitochondria permeabilization (middle line) and caspase-3 activation (bottom line) in the presence/absence of PARP inhibitors. Immunofluorescence with anti-activated-BAX (6A7) antibody (red), anti-cleaved caspase-3 (green) or live cells staining of the mitochondrial mass (MTG, green) and mitochondria with high transmembrane potential, mtΔΨ (MTR, red). Cell nuclei are stained with Hoechst (blue). The scale bars of BAX, caspase-3 and MTG/MTR are 50, 50 and 20 µm respectively. (**D**) PARP inhibition increases ADT-induced DNA damage. Comet assay of ADT-treated LNCaP cells ± OLA: the histograms report the Damage Index at the indicated time points. (**E**) The histogram shows the scores (from 0 to 4) of DNA damage of ADT-treated LNCaP in presence/absence of OLA at the indicated time point. Statistical significance was calculated via Student’s *t*-test. * *p* < 0.05, ** *p* < 0.01 and *** *p* < 0.001, with respect to ADT-treated cells.

We observed that 3-ABA and OLA do not cause any toxic effect on the cell viability of untreated LNCaP, apart from a slight temporary reduction in the proliferation rate (Figure 4A), which coheres with what was found in [22]. Instead, both PARP inhibitors exert a strong toxicity in ADT-treated LNCaP (Figure 4A), leading to culture extinction shortly after 10 days of ADT. This corresponds to an about 3-fold increase in the extent of apoptosis at T10 by both PARP inhibitors (Figure 4B). Notably, as shown in Figure 4C, 3-ABA and OLA strongly increase the fraction of cells: (a) highly positive for BAX activation (top line), (b) displaying total mitochondria permeabilization (middle line, note green vs. yellow/orange cells), and (c) strongly positive for caspase-3 activation (bottom line). Such alterations, to a level coincident with that of full apoptosis (see positive controls in Figure 2), occur in scattered cells, suggestive of an asynchronous process. As far as DNA damage is concerned, OLA enhances the comet signal to a level comparable with that of full apoptosis (Figure 4D, compare red vs. blue bars). Development of the comet analysis separating the classes of damage at the T10 time point, shows that PARPi totally changes the pattern of DNA damage distribution (Figure 4E), shifting the prevalent category from class one (mild damage) to class three (high damage), with many cells present in class four (compatible with apoptosis). Therefore, the high damage of scattered cells is responsible for the increased damage score induced by PARP inhibition, confirming the hypothesis that cells escape from an unstable state in an asynchronous way.

### 2.5. PARPi-Induced Apoptosis Is Caspase-Dependent

**Figure 5:**
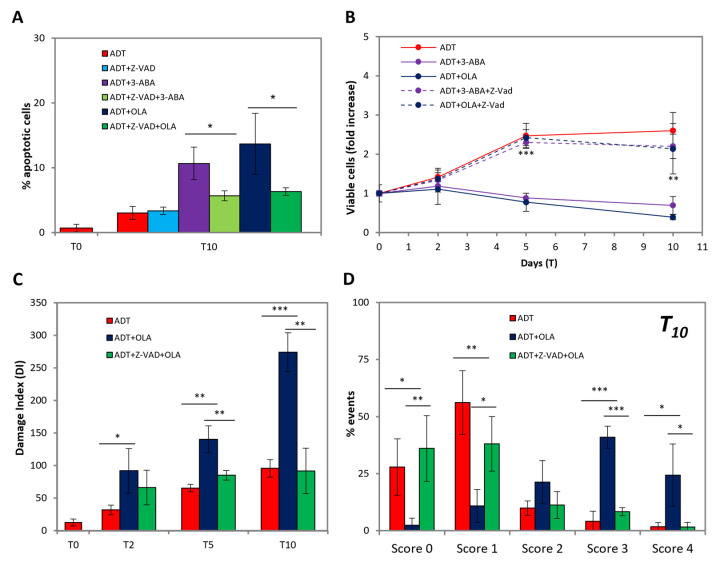
PARPi-induced apoptosis is caspase-dependent. (**A**) The increased apoptotic rate, induced by PARP inhibitors, is reverted by the caspase inhibitor Z-VAD: histogram shows the apoptotic extent at the indicated time points. Statistical significance was calculated via Student’s *t*-test. * *p* < 0.05. (**B**) Caspases inhibition reverts the cytotoxic effect of PARPi on ADT-treated cells. The profile shows the kinetics of viable (trypan blue-excluding) cell number expressed as fold over T0. Statistical significance was calculated via Student’s *t*-test. ** *p* < 0.01 and *** *p* < 0.001, with respect to ADT-treated cells + 3-ABA/OLA. (**C**,**D**) Caspase inhibition reverts PARPi-induced DNA damage, increasing the fraction of cells with low or null DNA damage. Comet assay of ADT-treated LNCaP cells + OLA ± Z-VAD. The histograms report the Damage Index (**C**) or the percentage of events in each damage categories (**D**) at the indicated time points. Statistical significance was calculated via Student’s *T*-test. * *p* < 0.05, ** *p* < 0.01 and ** *p* < 0.001.

We then investigated the role caspases play in PARPi-induced apoptosis. Figure 5A shows that at T10, i.e., when PARPi-induced apoptosis becomes evident, z-VAD significantly and strongly reduces apoptosis. Notably, z-VAD does not alter apoptosis induced by ADT alone, suggesting that an alternative, caspase-independent form of programmed cell death may intervene, e.g., parthanathos, which runs through the PARP-AIF-endoG axis [25]; this is coherent with the finding that at T10 z-VAD only partially reduces DNA damage (see Figure 3). Accordingly, z-VAD prevents the loss of cell viability due to PARP inhibition (Figure 5B), totally abrogating the vulnerability to PARP inhibition achieved by ADT.

Importantly, z-VAD reverts the extent of DNA damage with the PARP inhibitors to the value of ADT, both as total extents (Figure 5C) and size distribution (Figure 5D), indicating that all the extra damage induced by PARP inhibitors depends on caspase activation.

All this implies that PARP is required to stabilize viability in cells with a partially activated intrinsic apoptotic pathway, uncovering previously hidden targets of caspases responsible for bursting into full apoptosis, and providing an unexpected possible interpretation to the vulnerability to PARPi created by ADT [22].

### 2.6. ADT-Induced Quasi-Apoptotic State Can Be Reverted by Androgen Restoration

**Figure 6:**
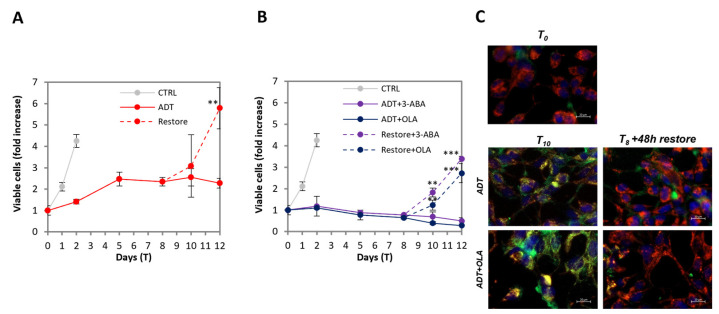
ADT-induced quasi-apoptotic state can be reverted by androgen restoration. (**A**) LNCaP cells treated with ADT for 8 days resume proliferation after androgen restoration. The profile shows the kinetics of viable (trypan blue-excluding) cell number expressed as fold over T0. Dashed line indicates androgen restoration. (**B**) Androgen restoration implies insensitivity to PARPi. The PARP inhibitors 3-ABA and OLA have no inhibitory effect on the proliferation of the rescued cells. The profile shows the kinetics of viable (trypan blue-excluding) cell number expressed as fold over T0. Dashed lines indicate androgen restoration. Statistical significance was calculated via Student’s *T*-test. ** *p* < 0.01 and *** *p* < 0.001, with respect to ADT-treated cells. (**C**) LNCaP cells treated with ADT ± OLA for 8 days can resume regular mitochondrial activity (red pattern) after 2 days of androgen restoration. Live cells staining of the mitochondrial mass (MTG, green), mitochondria with high transmembrane potential, mtΔΨ (MTR, red) and cell nuclei (Hoechst, blue) in wide-field micrography. The scale bar corresponds to 20 µm.

To investigate whether the viability of ADT-treated cells with DNA damage is a pre-apoptotic state preceding cell death, or a reversible feature, we re-administered the regular androgen level at T8 of ADT. We observed that, in the same guise as LNCaP collectively develop partial apoptotic features upon ADT, after androgen re-administration they collectively lose them and resume proliferation: the time course of viable cell number shows that after 48h there is already a slight increase, which becomes consistent at 96h, when cells have a proliferation rate approaching that of untreated cells (Figure 6A). We also analyzed ADT-treated cells in the presence of PARP inhibitors, followed by restoration of androgen though maintaining PARPi: also in this case, the culture is able to resume proliferation. The effect is even more substantial in this case, because it consists of the resumption of proliferation plus the stopping of cell loss (Figure 6B). Notably, in this case, the reversion also implies the loss of sensitivity to PARPi, which ceases to exert any inhibitory effect on the survival or proliferation of the rescued cells.

The return to proliferation is associated with the rapid reacquisition of regular mitochondria activity, as shown in Figure 6C, independently of the presence of PARPi: the yellow/orange, low-potential mitochondria have almost disappeared after as low as 48 h from androgen restoration, re-acquiring the red staining present in untreated LNCaP. Notably, the reversion to the previous state is collectively achieved by all cells that are “healed” by androgen resumption, and not due to the survival of a subset of cells that proliferate to replenish the culture.

The restoration of normal androgen levels that allows cells to resume proliferation was reported [26]; however, to our knowledge, it was never reported that this occurs by thorough single-cell healing. This implies that the metastable quasi-apoptotic state (QUAPS) may play an important role in ADT-promoted progression to CRPC.

### 2.7. LNCaP Recovered from ADT Are Genetically Unstable

ADT-promoted HSPC progression to CRPC in patients implies the induction of HSPC cells neo-proliferation, either because of androgen ectopic synthesis, or through the acquired ability of cells to proliferate in the absence of androgen. Notably, the protocol of androgen re-administration after ADT that we applied, mimics novel ectopic androgen synthesis, providing a system where the status of cells recovering after hormone restoration can be tested. Since it is not obvious why this should lead to the progression of HSPC to CRPC, we reasoned that the ADT-induced DNA breaks may hamper genetic integrity, thereby promoting progression, especially considering that ADT promotes strong DNA damage. 

**Figure 7:**
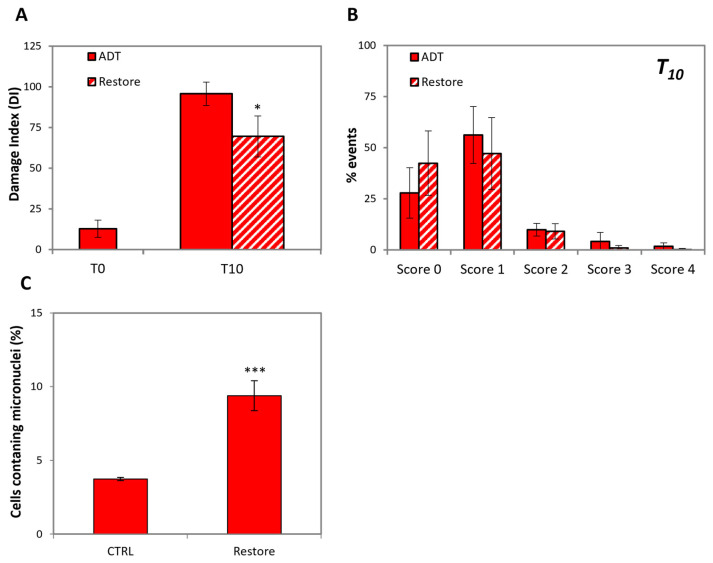
LNCaP cells recovered from ADT are genetically unstable. (**A**) LNCaP cells recovered from a 10-day treatment of ADT only gradually reduced DNA damage. Comet assay of ADT-treated LNCaP before and after androgen restoration: the histogram reports the Damage Index at the indicated time points. (**B**) The histogram shows the scores (from 0 to 4) of DNA damage of ADT-treated LNCaP before and after androgen restoration at the indicated time point. (**C**) LNCaP 48 h after androgen restoration show an increased extent of micronuclei. Statistical significance was calculated via Student’s *t*-test. * *p* < 0.05 and *** *p* < 0.01, with respect to control cells.

Figure 7A,B shows that, unlike mitochondria functionality that is rapidly restored by androgen re-administration, DNA repair seems to proceed more slowly, since the extent of DNA damage is still relatively high after 48 h, i.e., when cells already resumed proliferation.

The evidence that cells replicate in spite of severe DNA damage poses the problem of genetic integrity. To approach this issue, we performed a micronucleus assay on LNCaP 48 h after androgen restoration. Figure 7C shows that indeed, the extent of micronuclei is increased by about 3-fold, thus possibly causing mutagenesis upon further proliferation.

This provides a possible explanation for the fact that cells resume proliferation due to ectopic androgen production progress to CRPC with an increased mutation rate.

## 3. Discussion

In this study, we show for the first time that hormone-sensitive prostate cancer cells placed in androgen-deprived medium collectively enter a metastable quasi-apoptotic state (QUAPS) that includes caspase activation and the formation of dsDNA breaks. QUAPS may either progress into overt apoptosis upon the inhibition of PARP, or cells may heal and resume cycling upon androgen re-administration. QUAPS development encompasses all cells, suggesting that it is the result of a signal induced by hormone withdrawal rather than a sign of damage. Likewise, the return to the previous state is a collective reaction. Instead, the PARPi-induced overt apoptosis is an asynchronous occurrence, reminiscent of the induction of stress-induced apoptosis.

Cells in QUAPS express senescence features; since both senescence and QUAPS encompass practically all cells in ADT, the two conditions may overlap. Indeed, mitochondrial dysfunctions are often considered hallmarks of senescence [27,28]. This implies that upon ADT reversal, cells can escape from senescence. Senescence is a cell response to DNA damage, either as telomere dysfunction as in replicative senescence, or upon DNA breaks formation as in premature senescence [29]. Our finding that ADT induces DNA damage provides an explanation for the reported ADT-induced senescence [9].

Recently, it has been reported that cells can revert to the normal state after having passed through all steps of the intrinsic apoptotic signaling, provided the alterations were below a critical threshold: this process is known as “anastasis”, from the Grecian “coming back to life” [30]. The simil-apoptotic alterations may include caspase-induced DNA damage [31], which may induce genetic instability in the recovering cells [32]. Anastasis occurs in few scattered cells among a population undergoing full apoptosis after strong cytotoxic treatment [33], as the result of survival pathways acting at multi-levels after each step of the apoptotic signaling, including DNA endo-digestions. The reversion of QUAPS upon androgen restoration may in some way be reminiscent of anastasis, even though in this case, cell recovery is the result of a set of events put in motion by androgen receptor-mediated signaling, rather than the setup of survival pathways, as in anastasis.

QUAPS is a metastable state occurring as a programmed event that cells setup in response to non-cytotoxic changes in cell-to-cell communication within tissue, such as those induced by ADT. Importantly, QUAPS occurs in all cells as a collective reaction. To our knowledge, no such phenomenon has been described so far. All the evidence in our experimental observations point to QUAPS as a pro-survival measure to allow (cancer?) tissues to survive the homeostatic changes that follow androgen deprivation. It is not obvious why cells should self-inflict such severe alterations including, counterintuitively, caspase-mediated DNA endo-digestion to achieve survival. Our data indicate that QUAPS is a metastable state that requires PARP activity in order not to degenerate into apoptosis, thus preventing tissue collapse. It is known that PARP performs anti-apoptotic functions, which are generally attributed to the role it plays in DNA repair [34]. However, PARP exerts multiple functions on chromatin, deriving from its catalytic activity. Upon DNA damage, PARP is activated and promotes on chromatin the turnover of DNA binding proteins, which are repulsed from the negatively charged DNA after they are modified by PARP through the addition of long poly(ADP-ribose) chains, which are also negatively charged. This allows other proteins to access DNA, promoting multiple rounds of chromatin reshaping until the DNA damage is repaired [24]. It is therefore tempting to speculate that PARP activity may allow cells to manage the DNA breaks induced in QUAPS by keeping at bay the degeneration toward full apoptosis, at the same time assuring sufficient chromatin plasticity to achieve the necessary changes in gene expression required by the homeostatic changes that are occurring. At this regard, recently non-apoptotic functions of caspases have been reported [35], including the regulation of gene expression through the activation of the CAD endonuclease: for example, caspase-3-activated CAD produces DNA breaks at specific sites to promote myocyte differentiation [36], and in hematopoiesis, caspase may promote neutrophil differentiation [37].

How does QUAPS originate? Considering the canonical apoptotic signaling, the translocation of active BAX to the mitochondria causes mitochondrial permeabilization and ultimately caspase activation [18]. Here, we observed BAX activation and partial loss of mtΔΨ as early events of ADT, occurring in all cells just after hormone withdrawal. This suggests that the initial events of QUAPS occur in a similar way to regular apoptosis. If the events leading to QUAPS look quite straightforward, the primum movens is not yet identified. In apoptosis, BAX is activated by members of the BH3-only family or by redox alterations [38]. In ADT, it is consistently reported that low or null androgen levels are associated with mitochondrial dysfunction [39]. In the prostate of mHSPC patients undergoing ADT or ARi, mitochondrial deficit was observed in the normal and cancerous regions of the prostate [40], and also in other organs [41]. Interestingly and complementary, the recovery of mitochondrial functions in ADT through the upregulation of PPAIA4 is associated with the development of castration-resistance [42]. This is consistent with the knowledge that androgens exert beneficial effects on the mitochondrial function [43]. It is therefore possible to hypothesize a direct effect of ADT on mitochondria (or BAX?) as the primum movens of QUAPS. Detailed molecular/genetic/biochemical/metabolomic high throughput analyses, which are now in progress, will allow us to specifically identify the determinants of QUAPS [44,45] and the role played by PARP.

PARPi is now a standard therapeutic strategy in several cancers including ovary and breast carcinoma [46,47], on the basis of genetic pre-screening, for patients with mutant BRCA genes. In PC, it just became a standard option for the sub-population of CRPC patients bearing mutations in DNA repair genes [48,49]. However, it was experimentally proven in preclinical studies that PARPi, which is totally ineffective in the cell system used, is heavily synthetically lethal with ADT [22]. Our study confirms that ADT promotes vulnerability to PARP inhibition in HSPC cells, suggesting that PARPi co-therapy may also be applied for HSPC patients undergoing ADT, possibly even in the absence of DNA repair gene mutations [50], provided this is done before the emergence of androgen-independent growth, which may hinder PARPi-induced cytotoxicity. Scaling-up the complexity to 3D ex vivo samples [51] and in vivo studies will allow us to assess the clinical relevance of our results.

The mechanisms of ADT-induced progression from HSPC to CRPC are still poorly understood, limiting the possibility of developing strategies aimed at stabilizing the disease in ADT-treated patients. The results presented in this study, consisting of totally novel observations, may help focus the clinical management of ADT, contributing toward shedding light on the still obscure mechanism of ADT-promoted occurrences of castration-resistance, since DNA damage induced by ADT may easily be causative of genetic instability.

## 4. Materials and Methods

### 4.1. Cell Culture

Human mHSPC LNCaP and mCRPC PC3 cells (ATCC, Rockville, MD, USA) were grown in RPMI 1640 medium supplemented with 10% fetal bovine serum (FBS), 100,000 units/L penicillin, 50 mg/L streptomycin and 200 mM glutamine (Euroclone, Milan, Italy), at 37 °C in a humidified atmosphere of 5% CO_2_ in air and routinely split by trypsinization with Trypsin-EDTA (Euroclone, Milan, Italy). 

For all experiments, unless otherwise specified, LNCaP and PC-3 cells were seeded at a cell density of 17,500/cm^2^ and 12,500/cm^2^, respectively. Experiments are performed with a base-viability >98%.

Viable cell number was evaluated at selected time points following trypsinization and was assessed using a Burker counting chamber by the trypan blue-exclusion method.

### 4.2. Treatments

Androgen deprivation (AD): after 48 h from cell seeding, normal growth medium was removed and substituted with RPMI w/o phenol red supplemented with 2% charcoal-stripped-FBS (GE Healthcare, Chicago, IL, USA), 100,000 units/L penicillin, 50 mg/L streptomycin and 200 mM glutamine (Euroclone, Milan, Italy). AD media was replaced every 48–72 h. To assess the ability of AD-treated LNCaP to resume proliferation, after 8 days of treatment the AD media was replaced with normal media and the cell culture was monitored until confluence [52].

Apoptosis induction: the topoisomerase II inhibitor etoposide (Sigma-Aldrich, St. Louis, MO, USA) was used at the final concentration of 50 μM, as previously described in [44], and washed out after 48 h of treatment.

Caspase inhibition: pan caspase-inhibitor Z-VAD-fmk (Enzo Life Sciences, Farmingdale, NY, USA) was used at the final concentration of 10 μM.

DDR inhibition: ATM (Sigma-Aldrich, St. Louis, MO, USA) and ATR (AZD-6738, Cayman chemical, Ann Arbor, MI, USA) inhibitors were used at the final concentration of 10 μM and 1 μM, respectively.

PARP inhibition: 3-ABA (Calbiochem, San Diego, CA, USA) and Olaparib (Chemodex, St. Gallen, Switzerland) were used at the final concentration of 10 mM and 10 μM, respectively, the latter being clinically relevant [53].

All inhibitors were added together with normal or AD media, and replaced every 48–72 h. All treatments were administered to cells after 48 h from seeding or at 60% of confluence.

### 4.3. Senescence β-Galactosidase Staining

Senescence was assessed through the senescence β-galactosidase staining kit (Cell Signaling, Danvers, MA, USA). Briefly, cells grown in 6-well plates were washed with PBS, fixed for 15 min and stained with the β-galactosidase staining solution overnight at 37 °C in a dry incubator. Samples were then checked under a light transmission microscope (200× total magnification) for the assessment of blue color.

### 4.4. Micronuclei Assay

The micronuclei assay is a broad-spectrum mutagenesis test [54]. Micronuclei are small nuclear bodies arising from improper chromosome separation at mitosis as a consequence of mis-repaired DNA damage or chromosomal lost. The evaluation of the number of cells containing micronuclei among those undergoing the mitotic telophase is considered a measure of early mutagenesis.

To assess whether LNCaP cells resuming from ADT bear genetic instability, we trypsinized cells after a 5-day treatment of ADT, and seeded them in normal growth medium at a cell density of 12,500/cm^2^. Then, 24 h-post seeding, cytochalasin B (4 μg/mL; Sigma-Aldrich, St. Louis, MO, USA) was added to prevent cell division without inhibiting mitosis. After 24 h, the resulting bi-nucleated cells label those that underwent mitosis. The medium was then removed, the samples were rinsed with PBS, treated with hypotonic solution (KCl 0.075 M) for 3 min and then fixed by Carnoy fixative (methanol/acetic acid, 20:1) for 20 min. After washing with PBS, cells were stained with 2 μg/mL DAPI (Sigma-Aldrich, St. Louis, MO, USA) for 15 min, and then with Eosin (Sigma-Aldrich, St. Louis, MO, USA) for 1 min at room temperature. Samples were then rinsed with water and finally resuspended in PBS. 

Images were captured using a ZEISS Axio Observer (ZEISS, Oberkochen, Germany) microscope and analyzed using the Image J 2.0 software. 

The number of binucleated cells was evaluated on 1000 counted cells, whereas the number of cells containing micronuclei was evaluated on ≥300 bi-nucleated cells. Results were compared to those obtained for untreated LNCaP cells. 

### 4.5. Quantification of Apoptosis

Apoptosis was evaluated by quantifying the fraction of apoptotic nuclei by fluorescence microscopy (ZEISS Axio Observer, ZEISS, Oberkochen, Germany) after DNA staining with the cell-permeable specific dye Hoechst 33342 (Sigma-Aldrich, St. Louis, MO, USA) at the final concentration of 10 μg/mL, and was directly added to the cell culture. The fraction of apoptotic nuclei among the total cell population was calculated by counting 300 cells in at least three independent, randomly selected microscopic fields [13,55].

### 4.6. Immunofluorescence

#### 4.6.1. Vimentin

Samples grown over Nunc Lab-Tek chambers (Thermo Fisher Scientific, Waltham, MA, USA) were washed with PBS, fixed with 4% paraformaldehyde for 15 min, washed three times with PBS, permeabilized in PBS 0.3% Triton for 10 min and blocked with 1% BSA for 60 min. Samples were then incubated with primary antibody against vimentin (Sigma-Aldrich, St. Louis, MO, USA) in PBS 1% BSA for 1 h at room temperature. After three washes with PBS, samples were incubated with the FITC-conjugated secondary antibody (Sigma-Aldrich, St. Louis, MO, USA) at room temperature for 1 h. Samples were washed three times with PBS and cell nuclei were finally stained with DAPI (2 μg/mL). Images were captured using a ZEISS Axio Observer (ZEISS, Oberkochen, Germany) microscope and analyzed through the Carl Zeiss Microscopy GmbH’s ZEN 3.0 software.

#### 4.6.2. BAX-6A7, Activated-Caspase 3 and γH_2_AX 

Samples grown over Nunc Lab-Tek chambers (Thermo Fisher Scientific, Waltham, MA, USA) were washed with PBS, fixed with 4% paraformaldehyde for 15 min, washed three times with PBS and blocked with PBS 1% BSA-0.3% Triton for 60 min. Samples were then incubated overnight at 4 °C with primary antibody against BAX-6A7 (BD Bioscience, Franklin Lakes, NJ, USA), activated Caspase 3 (Cell Signaling, Danvers, MA, USA) or γH_2_AX (Cell Signaling, Danvers, MA, USA) in PBS 1% BSA-0.3% Triton.

After three washes with PBS, samples were incubated with the corresponding FITC-conjugated (Activated Caspase-3 or γH_2_AX) or TRITC-conjugated (BAX-6A7) secondary antibody (Sigma-Aldrich, St. Louis, MO, USA) at room temperature for 1 h. Samples were washed three times with PBS and cell nuclei were finally stained with DAPI (2 μg/mL). Images were captured using a ZEISS Axio Observer (ZEISS, Oberkochen, Germany) microscope and analyzed using the Carl Zeiss Microscopy GmbH’s ZEN 3.0 software.

#### 4.6.3. Mitochondria Analysis by Fluorescence Microscopy 

LNCaP were stained directly on 24-wells plates for 20 min with 100 nM MitoTracker^®^ Green FM (Thermo Fischer Scientific, Waltham, MA, USA), to mark the mitochondrial mass 100 nM MitoTracker^®^ Red FM (Thermo Fischer Scientific, Waltham, MA, USA), to detect the mitochondrial transmembrane potential (mtΔΨ) and 10 µg/mL Hoechst 33342 (Sigma-Aldrich, St. Louis, MO, USA) to stain cell nuclei. 

Images were captured using a ZEISS Axio Observer (ZEISS, Oberkochen, Germany) microscope and analyzed through the Carl Zeiss Microscopy GmbH’s ZEN 3.0 software.

### 4.7. DNA Damage Analysis by the Alkaline Comet Assay

Alkaline comet assay is a single-cell gel electrophoresis method that allows detection of both single and double DNA breaks on alkaline-denatured DNA. The experiment was performed according to protocols described in literature [56,57]. All reagents were purchased from Sigma-Aldrich, St. Louis, MO, USA.

At given time points, cells were detached by trypsin, centrifuged, resuspended in 0.5% low melting point agarose, distributed onto glass microscope slides pre-coated with a layer of 1% normal melting point agarose and finally, covered with coverslips.

After 10 min on ice, allowing gel solidification, coverslips were removed, and slides were incubated in the lysis mixture (pH 10) for 3 h in the dark. The lysis mixture was prepared adding 10% DMSO and 1% Triton X-100 to the lysis solution (2.5 M NaCl, 10 mM EDTA, 100 mM Tris-base and 0.2mM NaOH in deionized water).

After lysis, slides were rinsed for 15 min in the dark with alkaline running buffer (pH 13) to allow DNA unwinding. The alkaline buffer was prepared by dissolving 300 nM NaOH and 1 mM Na_2_EDTA in deionized water. Electrophoresis was conducted at 1 V/cm, 300 mA for 30 min in a unit Sub-cell GT System (15 × 25 cm) equipped with PowerPac™ HC High-Current Power Supply (Bio Rad Laboratories Inc., Hercules, CA, USA).

Slides were then gently washed for 5 min in the neutralization buffer solution at pH 7.5 (0.4 M Tris-base), washed in deionized water and left to dry in the dark at room temperature. Slides were finally stained with 50 µL of ethidium bromide (25 µg/mL) and analyzed by fluorescence microscopy.

Images were captured using a ZEISS Axio Observer (ZEISS, Oberkochen, Germany) microscope and analyzed using the Carl Zeiss Microscopy GmbH’s ZEN 3.0 software.

For each sample, 100 comets have been analyzed and divided into five damage categories (C_0_–C_4_), C_0_ being the least, as previously described [58]. The Index of Damage (ID) was calculated through the equation reported below:

ID = 0 × n°C_0_ + 1 × n°C_1_ + 2 × n°C_2_ + 3 × n°C_3_ + 4 × n°C_4_

n°C_x_ = number of cells in each category of damage.

### 4.8. Statistical Analysis

Each experiment was repeated ≥3 times. For data presenting, the mean ± SD was reported. Statistical evaluation was conducted by Student’s *t*-test (significance set at *p* < 0.05), using Past 4.06b software.

## Data Availability

Not applicable.
